# Factors responsible for long-term survival in metastatic breast cancer

**DOI:** 10.1186/1477-7819-12-344

**Published:** 2014-11-14

**Authors:** Keiichi Kontani, Shin-ichiro Hashimoto, Chisa Murazawa, Shoko Norimura, Hiroaki Tanaka, Masahiro Ohtani, Naomi Fujiwara-Honjo, Manabu Date, Koji Teramoto, Hitoshi Houchi, Hiroyasu Yokomise

**Affiliations:** Department of Thoracic, Breast and Endocrine Surgery, Kagawa University Faculty of Medicine, 1750-1 Miki-cho, Kita-gun, 761-0793 Japan; Department of Surgery, Japanese Red Cross Hospital, 4-1-3 Ban-cho Takamatsu, 760-0017 Japan; Department of Pharmacy, Kagawa University Hospital, 1750-1 Miki-cho, Kita-gun, 761-0793 Japan; Kagawa Health Service Association, Health Care Center, 148 Fuseishi, Takamatsu, 761-8071 Japan; Department of Radiology, Osaka Neurosurgery Hospital, 378-1 Sanmyo-cho, Takamatsu, 761-8083 Japan; Department of Surgery, Date Hospital, 588-8 Kanko-cho, 760-0076 Takamatsu, Kagawa, Japan; Department of Surgery, Shiga University of Medical Science, 1-1 Seta, 520-2191 Otsu, Shiga, Japan

**Keywords:** Metastatic breast cancer, Prognostic factor, Long-term survival, Metronomic chemotherapy

## Abstract

**Background:**

Although survival of patients with metastatic breast cancer (MBC) has been significantly prolonged over the past decade due to improvement of anti-cancer therapeutics, only a few patients survive for more than 10 years. It has not been determined which patients can have long-term survival with treatment.

**Methods:**

To determine prognostic factors responsible for long-term survival, we retrospectively compared clinicopathologic factors of patients with MBC who survived for 50 months or more after diagnosis with patients who did not. Of 70 patients with MBC who received chemotherapy between November 2005 and September 2011, 23 patients who survived for 50 months or more after diagnosis and 28 patients who died within 50 months after diagnosis were assessed for their clinicopathologic factors and outcomes.

**Results:**

The proportion of patients with hormone receptor-positive (HR+) tumors was significantly higher and the proportion of patients with triple negative tumors (TN) was lower in long-term survivors than in non-long-term survivors (HR+: 87% versus 28.6%, *P* = 0.000037; TN: 13.1% versus 53.6%, *P* = 0.0028). Metastatic site, number of disease sites, prior chemotherapeutic regimens and human epidermal growth factor receptor-2 (HER2) status did not differ between the two groups. The proportion of patients who received metronomic regimens was significantly higher in long-term survivors than in non-long-term survivors (65.2% versus 35.7%, *P* = 0.034) when the most effective regimen among regimens that were received in metastatic settings was compared between the two groups. Overall response rate was significantly higher (82.6% versus 17.9%, *P* <0.00001) and time to treatment failure after receiving the most effective regimen was longer in long-term survivors than in non-long-term survivors (26 versus 5 months, *P* = 0.0001). The number of chemotherapeutic regimens for breast cancer and that for MBC did not differ between the two groups.

**Conclusions:**

Patients with luminal-type MBC who benefit at least once from chemotherapy including metronomic regimens, or patients who continued to receive the most effective regimen for more than two years can be expected to have long-term survival after diagnosis of MBC, regardless of the number of chemotherapeutic regimens they had received.

## Background

Breast cancer has a relatively favorable prognosis compared with other cancers, such as lung, colon, ovarian and pancreatic cancers. Approximately 80% of women with primary breast cancer are expected to survive for at least 10 years after the operation (mastectomy or breast-conserving surgery) [1, 2]. However, of patients who have a relapse after the operation, or patients who were initially diagnosed with metastatic breast cancer (MBC), only about 5% survive for more than 10 years [3, 4]. Since women with MBC are unlikely to be cured from the disease, palliative therapy is taken into account for these patients for the purpose of prolongation of their survival, with maintenance of quality of life. There have been many clinical trials to assess the efficacy and safety of anti-cancer cytotoxic agents in metastatic settings of breast cancer [5–11]. In a first-line chemotherapy in this setting, both anthracyclines and taxanes are expected to have favorable activity, that is, response rates of 40 to 60% and survival of over two years, either by monotherapy or in combination with each other. In contrast, the efficacy of chemotherapy is not satisfactory in the second or later lines [12–19]. There are several possible reasons for these results, such as resistance to drugs, attenuated physical condition of the patients and less availability of drugs to use because of prior drug usage.

Although the aim of treatment for MBC is control of the disease and disease-related symptoms, treatment can occasionally achieve progression-free or disease-free long-term survival in patients with MBC [3, 20, 21]. Many factors are thought to be responsible for determining the survival term in MBC. Patient factors such as age, menopausal status, performance status, disease-free interval (DFI) and treatment that patients have received or are receiving are associated with prognosis. Further, tumor characteristics such as site of disease, number of disease sites, tumor grade, hormone sensitivity, human epidermal growth factor receptor-2 (HER2) status and other biological characteristics are likely to be responsible. However, the factors that are critical in predicting a patient’s survival remains to be determined. If factors involved in long-term survival of patients with MBC are identified, they would be helpful for physicians to make a decision regarding the choice of treatment strategy and avoiding ineffective and harmful interventions in daily clinical practice.

The aim of this study was to determine the factors responsible for long-term survival of patients with MBC. We retrospectively compared clinicopathologic features and clinical outcomes for patients with MBC who survived for a long period with those who did not survive for a long period.

## Methods

### Patients

Data for patients with advanced or recurrent breast cancer who were treated with chemotherapeutic regimens at Kagawa University Hospital between June 2006 and September 2011 were retrospectively analyzed in this study. The median age of the patients was 60 years (32 to 81 years), and the number of chemotherapeutic regimens received for MBC until patients were treated with the most effective regimen among those received in metastatic settings was three [1–7]. The median follow-up period was 30 months (1 to 68 months). The patients were divided into two groups according to their survival time after diagnosis of MBC: less than 50 months and 50 or more months. The former group, referred to as non-long-term survivors, included 28 patients who died within 50 months after diagnosis of MBC. There was no patient who died from other causes, such as other diseases, adverse events caused by treatment or accidents in this group, indicating that all of the patients died of MBC. The latter group, referred to as long-term survivors, included 23 patients who survived for at least 50 months after diagnosis, regardless of whether they were receiving anti-cancer treatment. There were 19 patients who were alive at the time of analysis but had not been followed up on for 50 months or more, and were not included in the comparison. Clinicopathologic factors and outcomes of the patients are shown in Table 1.

**Table 1:** **Comparison of clinicopathologic features of long-term survivors and non-long-term survivors**

	All patients	Patients with OS >50 months	Patient. with OS <50 months	***P***value
Number	70	23	28	
Age (years)	60 (32-82)	60 (37-82)	54.5 (32-81)	0.24
Disease-free interval (months)	36 (3-286)	60 (13-241)	13 (3-62)	0
Number of disease sites	2 (1-6)	2 (1-5)	2 (1-6)	0.56
Visceral lesion (%)	51.4	60.9	53.6	0.6
Tumor grade	2 (1-3)	1.5 (1-3)	3 (1-3)	0.07
Hormone-sensitive (%)	60	87	28.6	0
HER2-overexpressed (%)	18.2	22.2	25	0.84
Triple negative (%)	28.6	13.1	53.6	0
Prior A and T treatment (%)	35.7	47.8	35.7	0.39
Chemotherapy line	2 (1-5)	3 (1-5)	2 (1-4)	0.1
Chemotherapy line for MBC	1 (1-5)	2 (1-5)	1 (1-3)	0.03
Metronomic regimen (%)	45.7	65.2	35.7	0.03
Trastuzumab administration (%)	20	21.7	17.9	0.73

A and T, anthracycline and taxane; HER2, human epidermal growth factor receptor-2; MBC, metastatic breast cancer; OS, overall survival.

### Evaluation of therapeutic efficacy

Tumor responses were assessed by physical examination and computed tomography (CT), magnetic resonance imaging (MRI) or bone scan according to the Response Evaluation Criteria in Solid Tumors (RECIST) [22] every two to three months during treatment. Complete response (CR) was defined as the absence of evidence of disease, partial response (PR) was defined as a reduction in the product of the two largest perpendicular diameters of the target lesions by 50% or more, and progressive disease was defined as an increase in tumor size by 25% or more or the presence of a new lesion. Clinical response that did not meet any definition described above was classified as stable disease (SD). Clinical outcomes examined in this study included time to treatment failure (TTF), defined as the duration from initiation to discontinuation of treatment, time to progression (TTP), defined as the duration from initiation of treatment to disease progression or death of any cause, overall survival (OS), defined as the duration from initiation of treatment to death of any cause, and objective response rate (ORR). Toxicity was assessed according to the National Cancer Institute Common Toxicity Criteria, version 3.0 [23].

### Statistical analysis

We used the Mann-Whitney U test or standard chi-square procedures for comparison of the two groups. The effects of baseline characteristics, clinical responses or prognostic parameters on the risk of progression or death were assessed using Kaplan-Meier survival analysis and the log-rank test of significance. A 95% confidence interval (CI) for the median of each variable was computed using the method of Brookmeyer and Crowley [24]. We defined *P* <0.05 as significant; all *P* values were two-sided. SPSS statistical software system (SPSS Inc. Tokyo, Japan) was used for all calculations.

### Ethical consideration

This research was in compliance with the guidelines of the Ethics Committee at Kagawa University Hospital and conformed to the provisions of the Declaration of Helsinki in 1995.

## Results

### Baseline characteristics of patients

A comparison of baseline clinicopathologic features between the two groups showed that DFI was significantly longer in long-term survivors than in non-long-term survivors (60 versus 13 months, *P* = 0.003, Table 1). The proportion of luminal-type tumors was significantly higher and the proportion of triple negative tumors was lower in long-term survivors than in non-long-term survivors (luminal-type: 87% versus 28.6%, *P* = 0.000037; triple negative: 13.1% versus 53.6%, *P* = 0.0028). The proportion of patients who received metronomic regimens as the most effective regimen was significantly higher in long-term survivors than in non-long-term survivors (65.2% versus 35.7%, *P* = 0.034). Unexpectedly, the most effective regimen was administered in later lines in long-term survivors compared to non-long-term survivors (two versus one, *P* = 0.033), while the number of chemotherapy regimens for breast cancer was not different between the two groups (three versus two, *P* = 0.1). Other factors including age, number of disease sites, tumor grade, HER2 status and prior anthracycline and taxane administration were not different between the two groups.

### Efficacy of treatment and clinical outcomes

**Figure 1:**
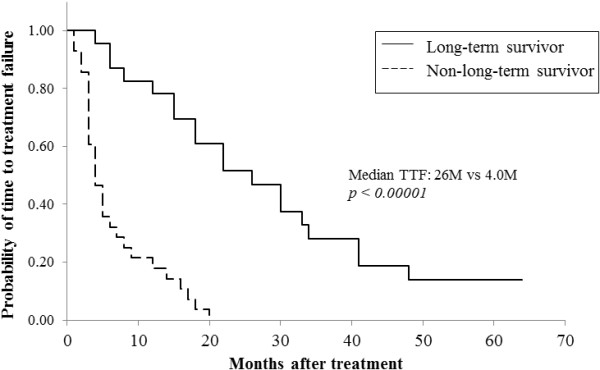
**Comparison of time to treatment failure (TTF) after the most effective regimen in long-term survivors and non-long-term survivors.**

**Figure 2:**
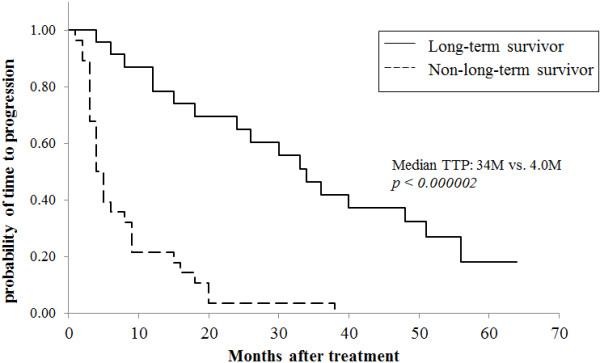
**Comparison of time to progression (TTP) after the most effective regimen in long-term survivors and non-long-term survivors.** PFS in the Figure 2 was changed into TTP.

**Figure 3:**
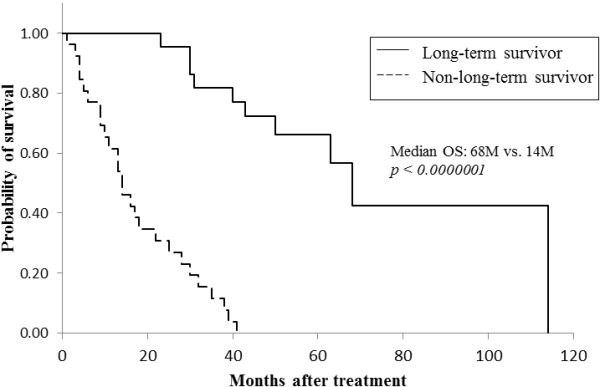
**Comparison of overall survival (OS) after the most effective regimen in long-term survivors and non-long-term survivors.**

A comparison of the clinical efficacy in the two groups showed that both objective response rate (ORR) and clinical benefit rate (CBR) were significantly higher in long-term survivors than in non-long-term survivors (ORR: 82.6% versus 17.9%, *P* <0.00001; CBR: 100% versus 35.7%, *P* <0.00001; Table 2). Of the 19 patients who showed tumor responses in the long-term survivors group, nine (26.1%) showed CR. In contrast, none of the non-long-term survivors showed CR (data not shown). To determine whether CR by the most effective regimen leads to prolongation of survival, we compared clinical outcomes of patients showing CR and patients not showing CR in the long-term survivors group. Although all of the outcomes including TTF, TTP and OS were longer in the CR group than in the non-CR group, there was no statistically significant difference between the two groups (Table 3). These findings suggest that tumor responses including long SD, PR and CR are needed in order to achieve long-term survival of 50 months or more, and that CR does not contribute to prolongation of survival. Furthermore, administration of one chemotherapeutic regimen for more than two years and also clinical benefit (no progression) lasting approximately three years obtained using one regimen were essential for long-term survival in MBC (TTF: 26 versus 5 months, *P* = 0.0003; TTP: 34 versus 5 months, *P* = 0.00013; Table 2 and Figures 1 and 2). The median survival time after receiving the most effective regimen was significantly longer in long-term survivors than in non-long-term survivors (68 versus 14 months, *P* <0.000001; Figure 3). The total number of chemotherapeutic regimens for MBC, for breast cancer, or after receiving the most effective regimen did not differ between the two groups (Table 2). Furthermore, when baseline characteristics were compared for patients who survived for 60 months or more and patients who died within 60 months after diagnosis of MBC as an exploratory analysis, the results were compatible with those described above (Tables 4 and 5).

**Table 2:** **Comparison of outcomes and administered regimens for long-term survivors and non-long-term survivor**

	All patients	Patients with OS >50 months	Patients with OS <50 months	***P***value
ORR (%)*	44.9	82.6	17.9	<0.00001
CBR (%)*	63.3	100	35.7	<0.00001
TTF*	10	26	5	0
TTP*	18	34	5	0
OS*	38	68	17	<0.00001
OS from diagnosis of MBC	52	113	20	<0.00001
Number Of chemotherapy regimens*	1 (0-3)	1 (0-3)	1 (0-3)	0.66
Number of chemotherapy regimens for BC	3 (1-7)	4 (1-7)	4 (1-6)	0.36
Number of chemotherapy regimens for MBC	3 (1-7)	3 (1-7)	3 (1-5)	0.2

*Responses, outcomes or chemotherapy regimens after the most effective regimen. BC, breast cancer; CBR, clinical benefit rate; MBC, metastatic breast cancer; ORR, overall response rate; OS, overall survival; TTF, time to treatment failure; TTP, time to progression.

**Table 3:** **Comparison of clinical outcomes of patients showing CR and those not showing CR**

	non-CR	CR	***P***value
TTF	20	34	0.22
TTP	30	51	0.29
OS	63	114	0.09
OS from diagnosis of MBC	95	103	0.78

CR, complete response; MBC, metastatic breast cancer; OS, overall survival; TTF, time to treatment failure; TTP time to progression.

**Table 4:** **Comparison of clinicopathologic features of long-term survivors (≥60 months) and non-long-term survivors**

	All patients	Patients with OS >60 months	Patients with OS <60 months	***P***value
Number	70	17	28	
Age (years)	60 (32-82)	60 (37-82)	56.5 (36-81)	0.86
Disease-free interval (months)	36 (3-286)	60 (13-241)	13 (3-62)	0.01
Number of disease sites	2 (1-6)	2 (1-3)	2 (1-6)	0.2
Visceral lesion (%)	51.4	52.9	50	0.91
Tumor grade	3 (1-3)	2 (1-3)	3 (1-3)	0.07
Hormone-sensitive (%)	60	88.2	28.6	<0.00002
HER2-overexpressed (%)	18.2	33.3	17.9	0.55
Triple negative (%)	28.6	5.9	60.7	0
Prior A and T treatment (%)	35.7	41.2	39.3	0.67
CT line	2 (1-5)	3 (1-5)	2 (1-4)	0.2
CT line for MBC	1 (1-5)	2 (1-5)	1 (1-3)	0.07
Metronomic regimen (%)	45.7	64.7	35.7	0.03
Trastuzumab administration (%)	21.3	23.5	16.7	0.41

A and T, anthracycline and taxane; CT, computed tomography; HER2, human epidermal growth factor receptor-2; MBC, metastatic breast cancer; OS, overall survival.

**Table 5:** **Comparison of outcomes and administered regimens for long-term survivors (≥60 months) and non-long-term survivors**

	All patients	Patients with OS >60 months	Patients with OS <60 months	***P***value
ORR (%)*	44.9	88.2	20	<0.00003
CBR (%)*	60	100	40	<0.00003
TTF*	10	26	5	0
TTP*	18	34	5	0
OS*	38	68	17	<0.000002
OS from diagnosis of MBC	52	113	20	<0.000001
Number of CT regimens*	1 (0-3)	1 (0-3)	1 (0-3)	0.97
Number of CT regimens for BC	3 (1-7)	5 (1-7)	4 (1-6)	0.34
Number of CT regimens for MBC	3 (1-7)	4 (1-7)	3 (1-5)	0.17

*Responses, outcomes or chemotherapy regimens after the most effective regimen. BC, breast cancer; CBR, clinical benefit rate; CT, computed tomography; MBC, metastatic breast cancer; ORR, overall response rate; OS, overall survival; TTF, time to treatment failure; TTP, time to progression.

## Discussion

Although the aim of treatment for MBC is control of the disease and disease-related symptoms, progression-free or disease-free long-term survival is occasionally observed following systemic treatment in patients with MBC [3, 20, 21]. However, who is expected to be a long-term survivor, or what strategy is the best for long-term survival, remains to be determined. In this study, we attempted to determine what prognostic factors are responsible for long-term survival by retrospectively comparing clinicopathologic features and clinical outcomes of patients with MBC who had survived for 50 months or more after diagnosis of MBC with patients with MBC who had died within 50 months after diagnosis. Of 70 patients with MBC who had received chemotherapy at our hospital between November 2005 and September 2011, patients who survived 50 months or more accounted for 38.6%, and patients who died within 50 months accounted for 40%. In terms of baseline clinicopathologic features associated with survival of the patients studied, the proportion of patients with longer DFI and the proportion of patients with hormone-sensitive tumors were significantly higher in long-term survivors, but the proportion of patients with triple negative tumors was significantly lower in long-term survivors (Table 1). Lower tumor grade seemed to show a trend for long-term survival, although there was no statistically significant difference between the two groups. In contrast, the number of prior chemotherapeutic regimens the patients had received was not lower in long-term survivors than in non-long-term survivors, and there was no relationship of metastatic sites, number of disease sites, prior chemotherapy regimens or chemotherapy line between the two groups. These findings suggest that long-term survival might be associated with slowly growing luminal A-subtype tumors.

As chemotherapy used in adjuvant settings, anthracyclines and taxanes are included in standard regimens because of abundant evidence from many clinical trials over the past several decades showing significant reduction in the risk of relapse or death from the disease [25–28]. In contrast, there is no standard regimen recommended in metastatic settings. In many cases of MBC, anthracyclines and taxanes had already been administered in adjuvant settings. Therefore, physicians often have difficulty in choosing a regimen among agents for which clinical studies have demonstrated their feasibility. In cases of life-threatening metastatic lesions or cases of rapidly growing tumors, regimens that are expected to control lesions quickly, such as taxanes in combination with either gemcitabine, capecitabine or bevacizumab, should be utilized [28–33]. However, many patients show progression of disease during or after receiving these cytotoxic regimens, even if favorable combinations are chosen. We previously demonstrated that long-term administration of one regimen was essential for favorable outcomes of treatment for MBC [34]. To prolong the duration of treatment or the TTF, chemotherapeutic regimens that are less toxic as well as effective are considered. Metronomic chemotherapy is defined as continuous or frequent treatment of low doses of anticancer agents, and is usually used for palliative care in patients who have been heavily pretreated with cytotoxic drugs, or patients who have poor performance status [35]. Interestingly, metronomic chemotherapy used for palliation has been reported to result in favorable tumor responses and prolonged survival in some cases [36–39]. In this study, the proportion of patients who received a metronomic regimen as the most effective regimen was two thirds of long-term survivors (65.3%), which was double that of non-long-term survivors (Table 1). Of 15 patients who were treated with metronomic regimens in the long-term survivors group, nine (60%) received the regimen for more than two years, and one had not received the regimen for two years but was continuing to receive the regimen at the time of analysis (data not shown). Metronomic regimens may therefore have greatly contributed to prolongation of the duration of treatment in this group.

The proportion of patients who showed favorable tumor responses to, or those who showed clinical benefit from, the most effective regimen in long-term survivors was three times (83%) and five times (100%) higher than in non-long-term survivors, respectively (Table 2). The median TTF was more than two years and TTP was approximately three years in long-term survivors. These data indicate that long SD or better tumor response (PR or CR) to treatment for MBC at least once and continuation of treatment with one regimen for more than two years are needed for long-term survival. TTP and OS after the most effective regimen were significantly longer in long-term survivors than in non-long-term survivors (Table 2, Figures 2 and 3). However, in contrast to our expectation, the number of chemotherapeutic regimens for breast cancer, for MBC or after receiving the most effective regimen did not differ between the two groups. Furthermore, to exclude the possibility that the prognostic factors described above resulted from a relatively short observation period in which the outcomes of the patients were compared, we performed an exploratory analysis comparing prognostic factors of patients who survived 60 months or more and patients who died within 60 months after diagnosis of MBC. As a result, all factors identified from baseline characteristics and clinical outcomes of the patients by the comparison did not differ from those that we compared at 50 months after diagnosis of MBC, except for number of prior regimens for MBC (Tables 4 and 5).

## Conclusions

Hormone-sensitive status is the most important factor as a baseline characteristic responsible for long-term survival. Furthermore, benefiting at least once from chemotherapy and continuation of treatment with one regimen for more than two years are needed for long-term survival.

## Abbreviations

CBR: Clinical benefit rate
CR: Complete response
DFI: Disease-free interval
ER: Estrogen receptor
HER2: Human epidermal growth factor receptor-2
MBC: Metastatic breast cancer
ORR: Overall response rate
OS: Overall survival
PR: Partial response
RECIST: Response Evaluation Criteria in Solid Tumors
SD: Stable disease
TTF: Time to treatment failure
TN: Triple negative
TTP: Time to progression.
